# Editor-in-Chief of the Structural Heart Journal: 1-Year Follow-Up

**DOI:** 10.1016/j.shj.2026.100835

**Published:** 2026-03-07

**Authors:** Josep Rodés-Cabau

**Affiliations:** a Quebec Heart & Lung Institute, Laval University, Quebec City, Quebec, Canada; b Clínic Barcelona, Barcelona, Spain

It was with great enthusiasm that, one year ago, I began my role as Editor-in-Chief of Structural Heart, the official journal of the Cardiovascular Research Foundation (CRF). This has been a tremendously rewarding personal and professional experience, and an enormous pleasure and honor to work with a dream team of Associate and Deputy Editors, engaging in multiple enriching discussions about manuscripts and new ideas for the journal, always striving to be fair and respectful on the evaluation of authors’ work. The contribution of our reviewers should also be highlighted, and I would like to express our sincere gratitude to them for their great work and invaluable contributions to the journal over the past months. Finally, the continued support and strategic guidance from the CRF organization have been essential to the journal’s stability and growth, with special mention of the outstanding work of Maria Alu, the Executive Editor and the true soul of the journal’s organization.

Several journal metrics and achievements from this past year also deserve special mention.

1. The number of manuscript submissions has increased by 80%. This growth has been driven primarily by a major increase in submissions from Europe and other regions outside the United States, along with a continued rise in US submissions. We are extremely pleased to see Structural Heart becoming a truly global journal, and we encourage authors from around the world to continue submitting their work.
2. We had a very successful and productive experience with the New York Valves and Transcatheter Cardiovascular Therapeutics meetings, with several high-quality manuscripts published simultaneously in our journal at the time of their meeting presentations. The opportunity for simultaneous publication will continue to be offered for abstracts presented at these two meetings—stay tuned.
3. Although aortic stenosis and transcatheter aortic valve implantation remain the main topics, we have observed a significant increase in submissions covering the full spectrum of structural heart disease, particularly mitral and tricuspid interventions, as well as several excellent manuscripts on left atrial appendage closure and other structural heart interventions. Expanding the scope of the journal beyond transcatheter aortic valve implantation and valvular interventions was one of our initial objectives, and we are clearly moving in the right direction.
4. In addition to high-quality original articles, we have published a series of high-level review articles covering diverse topics. Most of these reviews involved a junior fellow (often as first author) mentored by an experienced investigator. Indeed, the journal’s mentor–mentee program continues to attract significant attention. Overall, this strongly reflects the educational commitment of both the journal and the CRF organization.
5. The mean time from manuscript submission to first decision has been <20 days, with very few papers exceeding the 30-day target we set at the beginning of our term. We will continue to work tirelessly to further improve these timelines.
6. Investigators increasingly expect their work to be publicly available as rapidly as possible. I am pleased to announce that the turnaround time from manuscript acceptance to PubMed Central indexing has been significantly reduced, with a current mean time of about two months from acceptance.

We are living exciting times at Structural Heart. We will continue to work hard and rigorously to improve the journal’s quality and metrics, and we count on you—authors, reviewers, and readers—to join us on this exciting journey.

